# Anthropomorphism Index of Mobility for Artificial Hands

**DOI:** 10.1155/2019/7169034

**Published:** 2019-07-28

**Authors:** Immaculada Llop-Harillo, Antonio Pérez-González, Verónica Gracia-Ibáñez

**Affiliations:** Grupo de Biomecánica y Ergonomía, Departamento de Ingeniería Mecánica y Construcción, Universitat Jaume I (UJI), 12071, Spain

## Abstract

The increasing development of anthropomorphic artificial hands makes necessary quick metrics that analyze their anthropomorphism. In this study, a human grasp experiment on the most important grasp types was undertaken in order to obtain an Anthropomorphism Index of Mobility (AIM) for artificial hands. The AIM evaluates the topology of the whole hand, joints and degrees of freedom (DoFs), and the possibility to control these DoFs independently. It uses a set of weighting factors, obtained from analysis of human grasping, depending on the relevance of the different groups of DoFs of the hand. The computation of the index is straightforward, making it a useful tool for analyzing new artificial hands in early stages of the design process and for grading human-likeness of existing artificial hands. Thirteen artificial hands, both prosthetic and robotic, were evaluated and compared using the AIM, highlighting the reasons behind their differences. The AIM was also compared with other indexes in the literature with more cumbersome computation, ranking equally different artificial hands. As the index was primarily proposed for prosthetic hands, normally used as nondominant hands in unilateral amputees, the grasp types selected for the human grasp experiment were the most relevant for the human nondominant hand to reinforce bimanual grasping in activities of daily living. However, it was shown that the effect of using the grasping information from the dominant hand is small, indicating that the index is also valid for evaluating the artificial hand as dominant and so being valid for bilateral amputees or robotic hands.

## 1. Introduction

In recent years, it has been an increasing development of new affordable and anthropomorphic prosthetic hands [1, 2] as a consequence of the improvements in 3D-printing technologies. The human hand is a complex and marvelous tool whose dexterity has not been achieved by any artificial hand. Evaluating the functional similarity of artificial hands with the human hand is essential for improving current anthropomorphic hand designs. Assessing the capability of the prostheses to perform the main grasp types (GTs) of human grasping could give an insight into the level of functionality restored in patients. Metrics or indexes that quantify numerically the level of anthropomorphism are the way to grade human-likeness and to provide specifications for maximizing the anthropomorphic functionality while designing new artificial hands.

Belter et al. [1] reviewed and compared the mechanical properties of different prosthetic hands, as their degrees of freedom (DoFs), range of motion, and weight and number of actuators, but an index to compare those properties with the human hand was not defined. Some other previous studies tried to quantify the anthropomorphism of artificial hands with a numerical index. Feix et al. [3] proposed a metric for comparing the anthropomorphic motion capability of robotic and prosthetic hands, the anthropomorphism index (AI), being its computation cumbersome and based only on the position and orientation of the distal phalanges in different GTs. Liarokapis et al. [4] defined an anthropomorphism index to assess the robot's ability to mimic the human hand based on the comparison of the finger phalanx workspaces and also the workspaces of the fingers' base frames. Liu et al. [5] proposed twelve quantified prosthetic hand anthropomorphism evaluation indexes including physical and actuation properties, among which is included a DoF configuration evaluation. This index was based on a matrix of DoF configuration where the element of the matrix is set to 1 if there exist an artificial DoF in the corresponding position, otherwise is set to 0. However, this approach does not take into account the relevance of each DoF for grasping during activities of daily living (ADL) nor the underactuation in the joints. Underactuation in artificial hands [6] allows to use less actuators than DoFs while keeping versatility to adapt GTs to different object shapes.

Prostheses design could be different depending on its use for a dominant or nondominant hand; however, in the case of a patient who still has a healthy hand, the most appropriate strategy would be to consider the remaining hand as dominant [7, 8]. Thereby, the design of the prosthesis should be focused for a nondominant hand reinforcing bimanual grasping. The importance of the different GTs for personal autonomy of the patients in ADL has been studied previously by the authors [9], being pulp pinch (PP) (26%), extension grip (EG) (20.8%), tripod pinch (TP) (10.4%), and transverse volar grip (TVG) (8.7%), the most relevant GTs for a nondominant hand to reinforce bimanual grasping, representing together with the nonprehensile one, almost 90% of relevance for autonomy.

**Figure 1:**
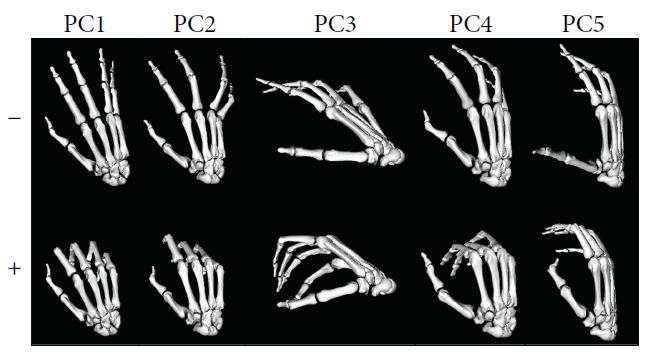
Principal components of the degrees of freedom of the human hand performing activities of daily living obtained in [10] (PC1: digit arching, PC2: closure, PC3: palmar arching, PC4: lateral pinch, and PC5: opposition).

In the previous studies by the authors [10], the posture of the right hand from 24 healthy subjects performing 24 representative ADL was recorded with an instrumented glove. ADL were selected from the WHO's International Classification of Functioning, Disability and Health [11]. By applying principal component analysis (PCA), five factors explaining 73.7% of the variance were obtained. As shown in Figure 1, the five main principal components (PCs) of the DoFs of the human hand in ADL were “PC1: digit arching” (flexion of the interphalangeal joints), “PC2: closure” (combination of abduction of the fingers, except for the thumb, with flexion of the metacarpophalangeal joints), “PC3: palmar arching,” “PC4: lateral pinch” (represents the lateral opposition of the thumb to the index), and “PC5: opposition” (represents the pad-to-pad opposition of the thumb to the little finger).

The aim of this study is to propose an index to measure the anthropomorphism of prosthetic hands, based on the comparison of the topology of the whole hand (joints and DoFs) and on the possibility to control these DoFs independently. The computation of the index, referred to as Anthropomorphism Index of Mobility (AIM), should weight each DoF depending on its importance for grasping in ADL. To define this importance, we used the information from previous experimental tests performed in the group and specific tests developed in this study on the main GTs. Furthermore, a preliminary study [12] carried out on four human healthy subjects encouraged us to go deep in the study by increasing the number of subjects, improving the definition of the index, and widening the analysis of its validity to the different types of artificial hands. The AIM is intended to be a quick computation index based on the biomechanics of the human hand and thus providing a way to compare their functional anthropomorphism. Moreover, the relevance of each DoF for functionality, obtained by tests on the human hand in this study, is intended to be useful for other applications in artificial hand design.

## 2. Materials and Methods

### 2.1. Human Grasp Experiment

With the purpose of taking into account in the AIM the relevance of each DoF according to its importance for functional grasping, an experiment to measure the kinematics of the human hand in functional grasps was carried out. Twenty subjects, ten males and ten females, all of whom were right-handed and free of hand pathologies or injuries, performed the most relevant GTs for a nondominant hand to reinforce bimanual grasping in ADL (PP, EG, TP, and TVG [9]). Although the grasps were selected for a nondominant hand (most common use of a hand prosthesis for unilateral amputees), subjects were asked to perform grasps with their dominant hand to get the most natural performance of human grasping. The study was approved by the Ethics Committee of the University, and all the subjects gave their written informed consent. The ages of the subjects ranged intentionally between 20 and 51, being the average 35 ± 8, in order to prevent kinematic alterations due to joint degeneration from ageing. Subjects were selected so that the distribution of hand sizes was representative of the population [13]. The hand width ranged from 70 to 96 mm with an average of 83 mm, and the hand length ranged from 170 to 210 mm with an average of 185 mm.

**Figure 2:**
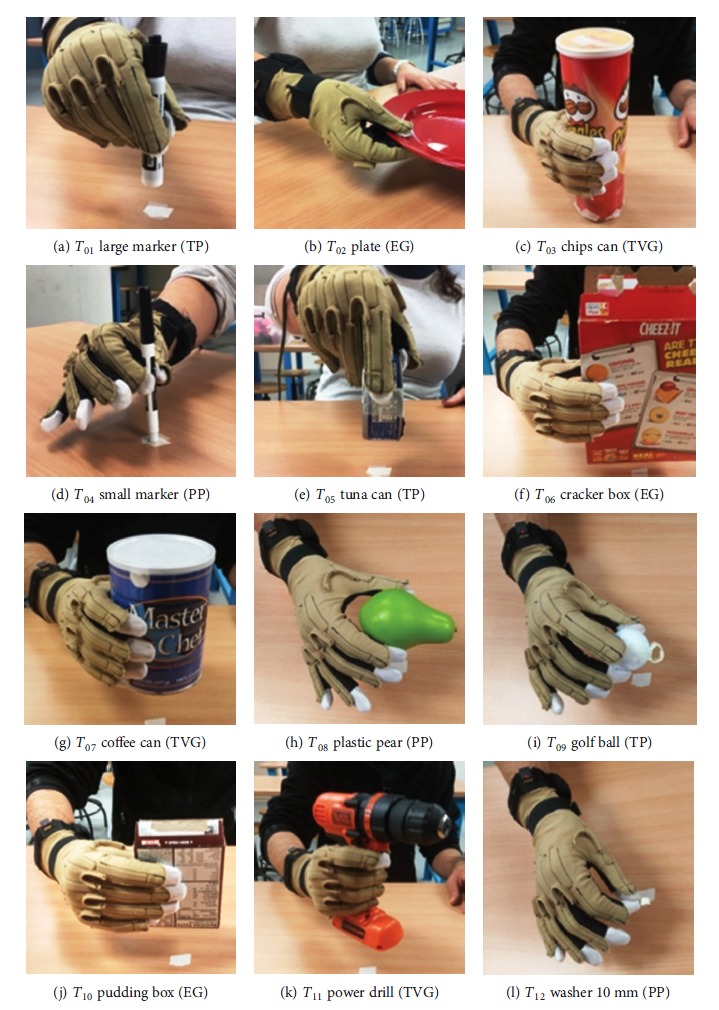
Grasping tasks of the experiment (a-l). *T*_*g*_ are the tasks ordered (*g*: indicates the order) followed by the object of the Yale-CMU-Berkeley Object and Model Set [14] to grasp and in brackets the grasp type to be performed in each task (TP: tripod pinch, EG: extension grip, TVG: transverse volar grip, and PP: pulp pinch).

Twelve objects of different sizes were selected from the Yale-CMU-Berkeley Object and Model Set [14], three for each of the four GTs (PP, EG, TP, and TVG), in order to cover most common requirements in ADL for each one (Figure 2). The subjects were sitting with the hands in the table in a comfortable way: the arms close to the body and parallel to the sagittal plane, the elbows flexed 90°, the wrist on the edge of the table, and the hands laying on the table palms down in a natural posture. This was the starting and ending posture for each grasping action. Subjects were instructed on the different GTs to perform with each object, and objects to be grasped were situated one by one by the researcher at a distance of 30 cm in front of the subjects. Subjects were free to practice the grasps to be sure that it is in the correct posture before starting the recordings. The steps to perform the grasps during the experiment consisted of the following: grasping the object from the table with the correct hand posture/GT, lift it up during two seconds, and finally, release the object again on the table and return the hand to the starting position. The sequence of the twelve objects to grasp during the experiment is shown in Figure 2 in the specified order. The experiment was repeated three times per subject.

The kinematics of the hand while performing the grasping postures was recorded (100 Hz) using an instrumented right hand glove with 18 sensors (CyberGlove Systems LLC; San Jose, CA). DoF kinematics corresponding to 16 joint angles (marked with an asterisk in Table 1) was obtained using a previously validated protocol [15]: metacarpophalangeal flexion (MCP1 to MCP5, 1 to 5 meaning thumb to little digits), interphalangeal flexion of the thumb (IP1), proximal interphalangeal flexion of the fingers (PIP2 to PIP5), flexion and abduction of the carpometacarpal joint of the thumb (CMC1), relative abduction between finger MCPs (index-middle, middle-ring, and ring-little), and palmar arching. Prior to the tests with objects, the CyberGlove was calibrated for each subject following the calibration procedure [15]. Starting and final positions while the hand is not moving were trimmed from the recordings. Then, they were filtered with a 2nd-order 2-way low-pass Butterworth filter with cut-off frequency of 5 Hz [16, 17]. The tests were video recorded.

### 2.2. Index Definition

The Anthropomorphism Index of Mobility (AIM) for an artificial hand was defined based on two main factors: (1) the DoFs present in the hand along with its method of actuation and (2) the relevance of these DoFs for grasping in ADL.

The DoFs of the human hand (HH) [18, 19] were classified into four different functional groups for defining the AIM (Table 1): finger flexion-extension (12 in HH), finger abduction-adduction (4 in HH), palmar arching (2 in HH), and thumb opposition (5 in HH).

The Anthropomorphism Index of Mobility (AIM) was defined with

(1) $$\begin{array}{c} \text{AIM}=\underset{i}{\sum }\left(k_{i}\cdot w_{i}\right), \end{array}$$

where the summation extends for *i* = 1,2,3,4, corresponding to each one of the four groups of DoFs (Table 1: F/E, AB/AD, P.ARC, and T.OPP), the factor *k*_*i*_ accounts for the type of actuation of the DoFs included in this group, and the factor *w*_*i*_ is a weighting coefficient depending on the relevance of this group of DoF for grasping in ADL. Both the term *k*_*i*_ and the weighting factor *w*_*i*_ were defined to have a range between 0 and 1, and the sum of weighting coefficients *w*_*i*_ for the four groups is unity, so that the AIM reach a maximum value of 1 for the human hand and a very low value for an artificial hand with very poor anthropomorphism.

The factor *k*_*i*_ for each group *i* was defined to get a high value if the method of actuation for the DoFs in that group allows to control them independently, as in the human hand, and a lower value if the motions of these DoFs are highly coupled during motion. To this end, each DoF in the evaluated hand was classified according to the types included in Table 2.

The independent mobility of a DoF can be ranked qualitatively from better to worse, depending on its class, as A>B>C>D>E. Note that B class was considered better than C because it allows mechanical adaptation of the finger to the shape of the object to be grasped and do not suffer from mechanical singular configurations [6]. Pugh's method used in concept design evaluation [20] was employed to convert the ranked list of methods of actuation of the DoFs into a list of numerical coefficients *c* (last column in Table 2). However, the independent mobility of a DoF is associated not only with the type of actuation in this particular DoF but also with that of the DoFs more proximal in the same serial chain of a digit, i.e., for a finger, the mobility for flexion in the PIP joint is dependent on the mobility for flexion in the MCP joint. Consequently, for that case, the coefficient *c*_*ij*_ for the DoF *j* of the group *i* was obtained as the multiplication of the coefficient *c* of this DoF and those located proximally in the same serial kinematic chain. In addition, for assigning the coefficient *c* to several DoFs underactuated by the same motor or actuator, class A was considered for only one of them and class B or C for others. If a motor actuates several DoFs included in different groups *i*, the coefficient 1 corresponding to class A was divided among the number of groups and this fraction was assigned to only one of the DoFs in this group, being others classified as either B or C. Finally, the factor *k*_*i*_ for each group *i* was defined with equation (2), by summing the terms *c*_*ij*_ in the group *i* and dividing by the number of DoFs of the human hand in this group (*n*_*i*_), which is, according to Table 1, 12 for *i* = 1, 4 for *i* = 2, 2 for *i* = 3, and 5 for *i* = 4.

(2) $$\begin{array}{c} k_{i}=\frac{\sum_{j}c_{ij}}{n_{i}}. \end{array}$$

The weighting factor *w*_*i*_ in equation (1), accounting for the relative relevance of the DoFs of the group *i* for grasping in ADL, was defined with

(3) $$\begin{array}{c} w_{i}=\sum_{k}\left(r_{ik}\cdot s_{k}\right). \end{array}$$

In equation (3), *r*_*ik*_ weights the relative contribution of the group of DoFs *i* (*i* = 1,2,3,4) in human hand functionality represented through PC*_k_* (*k* = 1,2,3,4,5), corresponding to each of the five kinematic functional synergies (see Figure 1) found in a previous study [10]. These PCs account for 73.7% of the variance when performing a wide set of representative ADL. The loading matrix of the PCs obtained in that study, which can be found in Supplementary Materials (available here), was used to calculate *r*_*ik*_ as shown in equations (4) and (5). For a PC*_k_*, *r*_*ik*_ was computed as the sum of absolute values of the loadings *l*_*ijk*_ for the DoFs *j* included in the group *i* (according to Table 1) divided by the sum of the absolute value of all the loadings of that PC*_k_*.

(4) $$\begin{array}{c} r_{ik}=\frac{\sum_{j}\left|l_{ijk}\right|}{a_{k}}, \end{array}$$

(5) $$\begin{array}{c} a_{k}=\sum_{i}\sum_{j}\left|l_{ijk}\right|. \end{array}$$

On the other hand, *s*_*k*_ in equation (3) contains the information about the importance of the PC*_k_* in the most relevant GTs. To compute this term, first, the human hand kinematics was obtained from the human grasp experiment explained above, but to consider the relation with the functionality of the human hand during ADL, kinematics was transformed to be expressed as scores *f*_*tk*_ referred to the five functional PCs (Figure 1) instead of being expressed in the original sixteen variables (joint angles). This information can be found in Supplementary Materials. A greater absolute value of the score of a PC*_k_* in one particular instant *t* indicates that the position of the hand is better represented by this PC*_k_*. Next, for each of the twelve grasping tasks *g* (Figure 2), the absolute value of the scores *f*_*tk*_ for each PC*_k_* was averaged during the task (equation (6)), and then (equation (7)) these means *v*_*gbp*_ were averaged across subjects *b* and repetitions *p*. The resulting means *v*_*g*_ were normalized (equation (8)) with respect to their sum across PCs *h*_*g*_ (equation (9)), providing the relative contribution of the five PCs to each grasping task *n*_*g*_. Finally, these relative contributions were weight-averaged by the relative relevance of the GT for autonomy of each grasping task *z*_*g*_ and divided by 3 because three objects were considered for each GT (equation (10)). The weight *z*_*g*_ for averaging was obtained from the relative use of the four main GT for a nondominant hand in bimanual grasping [9]: 39.5% for PP, 31.6% for EG, 15.8% for TP, and 13.2% for TVG.

(6) $$\begin{array}{c} {\left(v_{gbp}\right)}_{k}=\frac{\sum_{t=1}^{m}\left|f_{tk}\right|}{m}, \end{array}$$

(7) $$\begin{array}{c} {\left(v_{g}\right)}_{k}=\frac{\sum_{b}\sum_{p}{\left(v_{gbp}\right)}_{k}}{b\cdot p}, \end{array}$$

(8) $$\begin{array}{c} {\left(n_{g}\right)}_{k}=\frac{{\left(v_{g}\right)}_{k}}{h_{g}}, \end{array}$$

(9) $$\begin{array}{c} h_{g}=\sum_{k}{\left(v_{g}\right)}_{k}, \end{array}$$

(10) $$\begin{array}{c} s_{k}=\frac{\sum_{g}\left[{\left(n_{g}\right)}_{k}\cdot z_{g}\right]}{3}. \end{array}$$

### 2.3. Artificial Hands

With the objective of exemplifying the use of the AIM and verifying its utility, it was computed for several artificial hands with different topologies and actuation systems. The AIM was obtained for different affordable 3D-printed prosthetic hands, including the IMMA hand designed by the authors [21], some advanced commercial prosthetic hands, and other artificial hands. Some hands of these two later groups have been evaluated with other indexes of anthropomorphism in the literature, such as the anthropomorphism index (AI) [3] and the Total Score of Anthropomorphism (*A*_*R*_) [4]. The main characteristics of the hands analyzed are described below.

#### 2.3.1. Affordable 3D-Printed Prosthetic Hands

- IMMA hand [21]: 3D-printed five-digit prosthetic hand, with 6 DoFs actuated by tendons: flexion in each finger and flexion and abduction in the thumb. It has three phalanges per finger and its joints are elastic elements. This hand is just a prototype and cannot be used as a prosthesis directly, it needs a socket with motors and a control system, but after a study of the authors [22], it is being considered here to be actuated by two motors following the two actuation synergies obtained from experiments with human actuation
- Cyborg Beast [23]: five-digit low-cost 3D-printed prosthetic hand for children with upper-limb differences. It is body-powered using the wrist of the amputee as the unique actuator to drive all the finger tendons. It has two phalanges per finger and 5 DoFs: flexion in each finger and flexion of the thumb. Finger flexion is driven by tendons along the palmar surface of each finger. Elastic cords placed inside the dorsal aspect of the fingers provide passive finger extension. Its joints are Chicago screws and the materials used to print the different parts of the hand are PLA and ABS
- Flexy-Hand [24]: 3D-printed five-digit prosthetic hand, with 5 DoFs actuated by tendons: flexion in each finger and flexion in the thumb. It has three phalanges per finger and two phalanges in the thumb. The retraction is made through flexible 3D-printed joints. It is body-powered using the wrist of the amputee as the unique actuator to drive all the finger tendons
- KIT prosthetic hand [25]: five-digit 3D-printed hand prosthesis with underactuated mechanism, sensors, and embedded control system, developed by the Institute for Anthropomatics and Robotics (Karlsruhe Institute of Technology). Two motors (one for the four long fingers and other for the thumb) actuate 10 DoFs (flexion of two joints in each finger) by means of tendons. The four long fingers are simultaneously driven via a force-distributing transmission based on the TUAT/Karlsruhe mechanism providing shape adaptivity (all fingers keep closing until contact regardless of blocked movement in other joints). The passive reopening of the fingers is obtained through custom made springs
- ADA [26]: Ada Hand V1.1 by Open Bionics is a five-digit myoelectric prosthetic hand entirely 3D-printed with flexible material. It is tendon driven and has two phalanges in each finger and one linear actuator for each digit driving their flexion

#### 2.3.2. Commercial Prosthetic Hands

- i-Limb: myoelectronically controlled, externally powered, tendon linking, multiarticulating prosthetic hand of Touch Bionics [27] with eleven joints (two joints in each long finger and three in the thumb). It has five individually powered digits and powered thumb rotation, with manual override
- Bebionic [28]: multiarticulating myoelectric prosthetic hand developed by RSL Steeper with eleven joints (two joints in each long finger and three in the thumb). It has five actuators, one for each finger, and the thumb has two positions manually placed by the user with an inbuilt sensor detecting the position. Folding links allow the fingers to flex. Among the 14 different grip patterns and hand positions that it can achieve, one of them is the finger adduction grip
- SensorHand Speed: the Ottobock SensorHand Speed [28] is a myoelectronically controlled hand with three actuated fingers, which are driven by the same motor. It is covered with a cosmetic glove that emulates the ring and little finger using a metal bar within the glove, which couples these fingers to the movements of the middle finger
- Michelangelo hand: five-digit technologically advanced prosthetic hand of Ottobock [28]. Actively driven elements are the thumb, index finger, and middle finger while the ring finger and little finger passively follow the other fingers. The six joints are controlled by two actuators (one for the flexion/extension of the five digits and the second one allows the thumb to be electronically positioned in an additional axis of movement being abducted/adducted). The fingers are slightly abducted when the MCP joints are extended, and when flexed, the fingertips adduct and touch each other, providing a finger abduction/adduction mode

#### 2.3.3. Other Artificial Hands

- FRH-4 hand: this is a robot hand built for the mobile-assisting robot ARMAR [29]. It has eight independent fluidic actuators: one in the metacarpus that allows the palm to flex in the middle, the index and middle fingers have two each, the ring and little fingers have one for both, and the thumb has two actuators
- Barrett [30]: three-digit programmable grasper of Robotnik. It has four brushless motors and three multijointed fingers (two phalanges connected by belt transmission), two of them have an extra DoF with 180° of lateral mobility
- DLR/HIT II [31]: used on Space Justin (humanoid upper body) for telemanipulation is a multisensory robot hand from Harbin Institute of Technology and DLR Institute for Robotics and Mechatronic. It has 15 DoFs, five identical modular fingers with two flat BLDC motors placed in the base. Each finger has three active DoFs (2 DoFs of flexion and one of abduction) and four joints (the motions of distal and medial phalanges are coupled by a linkage). The thumb is fixed in an appropriate orientation of the palm
- Shadow [32]: the Shadow Dexterous Hand is a humanoid robot hand created by the Shadow Robot Company. The four fingers have 2 one-axis joints (DIP and PIP) and one universal MCP joint; the little finger has an extra one-axis joint on the metacarpal to provide arching. The thumb has a one-axis joint (IP) and two universal joints (MCP and CMC). It contains 20 motors in the forearm (3 DoFs per finger, 5 DoFs in the thumb, 1 DoF in the palm, and 2 DoFs in the wrist)

## 3. Results and Discussion

**Figure 3:**
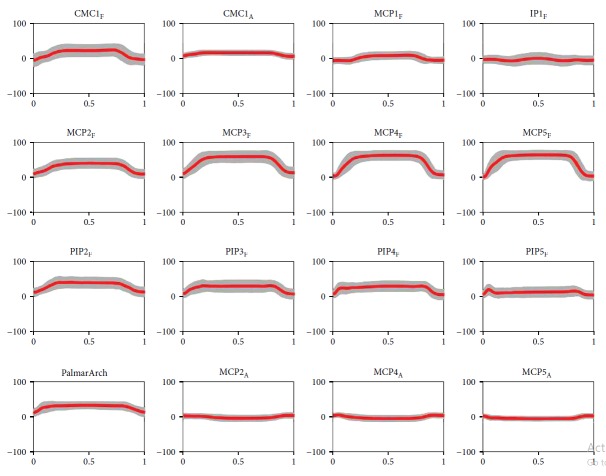
Mean and standard deviation of the 16 joint angles (in degrees) measured with the CyberGlove during the extension grip of the plate (*T*_02_). The angles are normalized across time (1: thumb, 2: index finger, 3: middle finger, 4: ring finger, and 5: little finger; CMC: carpometacarpal joint, MCP: metacarpophalangeal joint, PIP: proximal interphalangeal joint, DIP: distal interphalangeal joint, IP: interphalangeal joint; F: flexion/extension; and A: abduction/adduction).

Figure 3 shows a representative plot of the mean value and standard deviation across all the subjects (20) and repetitions (3) of the 16 joint angles measured with the CyberGlove while performing a grasp of one object of the human grasp experiment (*T*_02_ of Figure 2).

Table 3 shows the relative contribution of the five PCs to each grasping task of the human grasp experiment (*n*_*g*_)_*k*_ (equation (8)) together with the final value of the parameter *s*_*k*_ for each PC (equation (10)), obtained from these relative contributions weight-averaged by their importance in ADL *z*_*g*_ [9].

The contributions of the different PCs to each grasping task ranged between 9.6% and 39.1%, indicating that all the five PCs have a nonnegligible importance in the twelve grasping tasks analyzed. It can be seen that PC1, corresponding to “digit arching,” is predominant in grasping tasks involving PP (*g* = {4,8,12}). Moreover, “palmar arching” (PC3) and “lateral pinch” (PC4) synergies are less represented in average in the four main GTs considered in the experiment, leading to lower values of the final parameter *s*_*k*_ for these PCs, although the difference with “opposition” (PC5) is small. “Digit arching” (PC1) is the most significant synergy, as indicated by the higher value of *s*_*k*_.

Table 4 shows the matrix *r*_*ik*_ (equation (4)) containing the influence of the different groups of DoFs of the human hand on the five kinematic functional synergies (Figure 1) and the resulting parameter *w*_*i*_ after applying equation (3) with this matrix *r*_*ik*_ and the vector *s*_*k*_ (Table 3).

The parameter *w*_*i*_ weights the relevance of the different groups of DoFs in the human grasps in ADL. These results indicate that finger flexion-extension is by far the most relevant group of DoFs accounting for more than half of the functionality, followed by thumb opposition and finger abduction-adduction. The palmar arching has a relevance of only 5%. This result by itself is valuable for making decisions during the design of new artificial hand prototypes to maximize their functionality.

Table 5 shows the value of the parameter *k*_*i*_ (equation (2)) for each group of DoFs for the artificial hands analyzed. The details about the computation for each hand (*c*_*ij*_) are supplied as Supplementary Materials. It can be seen that F/E and T.OPP are the groups of DoFs mainly included and actively driven in the artificial hands, manifested by higher values of *k*_*i*_. It is worth to note that this fact is coherent with the greater relevance of these groups of DoFs in ADL, as indicated by the parameter *w*_*i*_ (Table 4). Notwithstanding, some hands as the SensorHand and Michelangelo showed low scores in F/E because of their rigid fingers without interphalangeal joints. The unique hand with the 5 DoFs in T.OPP actively driven is the Shadow hand. AB/AD is included actively in DLR/HIT II and Shadow hands and passively through the use of deformable joints in some 3D-printed hands. Finally, P.ARC is only present in FRH-4 and Shadow hands.

Finally, Table 6 shows the AIM for the different artificial hands, obtained using equation (1) and considering the parameters shown in Tables 4 and 5. Two factors affect the final AIM obtained by a hand (equation (1)): its mobility and type of actuation, represented by the number of DoF, the number of actuators, the number of digits and phalanges per digit, and the type of underactuation, affecting to the final parameters *k*_*i*_; and how this mobility and actuation system is distributed among the different groups of DOFs, with regard to the human hand, affecting through the weighting factor *w*_*i*_ (Table 4). The most advanced robotic hands (DLR/HIT II and Shadow) with a significant amount of motors and DoFs, and located in the important groups of DoFs, with higher weight *w*_*i*_ (F/E, T.OPP, and AB/AD), obtained the highest AIM scores, above 75%. The commercial prosthetic hands i-Limb and Bebionic as well as some 3D-printed hands (ADA, IMMA) obtained AIM scores between 40% and 50%. These hands include a reasonable number of motors and DoFs in the important groups (F/E and T.OPP). The rest of the hands obtained scores below 40% with the lowest score being for the SensorHand. The reason behind this lower AIM is an improvable number of DoFs, motors, or type of underactuation in the groups of F/E, T.OPP, or both.

The results shown in Table 6 indicate that the artificial hands analyzed in the literature with other anthropomorphism indexes, such as AI [3] or *A*_*R*_ [4], are ranked equally by the AIM and the other metrics, although the scores are different. The method used to compute the indexes justify these different scores. The AI is obtained from the achievable workspace of positions and orientations of the fingers' distal segments and compares this with information obtained experimentally from human hand grasping. The *A*_*R*_ is based on the computation of the finger phalanx workspace combined with that of the finger base frames, and the comparison with the human hand is made through a simplified model of their joints and geometry. It is worth to note that obtaining AI and *A*_*R*_ involves using complex algorithms and detailed information of the hand design, not easily available, while obtaining the AIM just requires information about the number of DoFs and the possibility to control them independently. Despite these differences in the method used to obtain each index, the fact that they rank equally, the hands as the AIM can be seen as a kind of validation of our index. Two main points can justify the use of the AIM as a method for evaluating the anthropomorphism of an artificial hand.

- It is really quick to obtain: simply, the parameter *k*_*i*_ has to be calculated, according to the DoFs and actuation methods of the artificial hand and equation (1) has to be applied (*w*_*i*_ is provided above)
- It analyzes not only the topology but also the functionality of the artificial hand because it takes into account the results obtained in grasping tests and ADL with the human hand

Notwithstanding, some important aspects in the design of an artificial hand are not within the scope of the AIM: the orientation of the joint axes, the range of motion of the different hand joints, the dimension of the phalanges, the friction coefficient of the parts of the hand in contact with the objects, the grasping force exerted by the actuators, the efficiency of the driving linkages, the control system, etc. Some previous studies [33–35] have shown the relevance of these aspects. In this sense, the AIM, involving mainly the topological structure, the number of actuators, and the type of underactuation, can be considered as an index especially useful in the concept design stage. The other design considerations cited above should be taken into account in later design stages: preliminary or detail design. Additional indexes that take into account these aspects could be interesting, and future works can go in this way. The index proposed by Liu et al. [5] considers some of these aspects, but it does not include their relevance for functionality according to human grasping tests. With respect to the phalanx dimensions and the joints' range of motion, the authors developed some studies [13, 16, 36] helping to obtain anthropomorphic designs. However, the evaluation of some of the design aspects cited above is difficult to be performed with indexes, requiring experimentation, after detailed design of the artificial hand and manufacturing a prototype. The authors have proposed methods for this experimental evaluation considering the main GTs in ADL and a special device for actuating the hand prototype [21].

The ranges of motion of the hand joints obtained in the human grasp experiment undertaken in this study are shown in Table 7. A wide range of motion for the different joints was covered with the objects selected in comparison to the functional range of motion of the human hand joints in ADL [16]. These ranges could be considered as a minimum for prostheses with functional grasping for the main GTs, although general manipulation would recommend using larger ranges if possible.

This study was primarily focused on prosthetic hands, and therefore, the scoring system takes into account the capability of the hand to perform the most important GTs for a nondominant hand to reinforce bimanual grasping (through parameter *z*_*g*_). For the case of a dominant hand reinforcing bimanual grasping, the parameter *z*_*g*_ for the four GTs considered in this study changes to [9] PP (58.0%), EG (16.6%), TP (9.5%), and TVG (16.0%). The effect of this change on the resulting *w*_*i*_ is negligible and implies a disparity of the AIM obtained for the artificial hands analyzed (Table 6) of a maximum of 1%. Therefore, the AIM is considered useful to evaluate the anthropomorphism of both dominant and nondominant hands. With this result and the result obtained from the comparison of the AIM with other indexes of the literature [3, 4], we can conclude that the index proposed can be valid for artificial both robotic and prosthetic hands, regardless of whether they are dominant or nondominant hands.

## 4. Conclusion

In this study, we have presented an anthropomorphism index (AIM) that can be used to evaluate and compare the mobility of artificial hands in relation to the human hand functionality, especially in concept design. The AIM evaluates the topology of the whole hand (joints and DoFs) and the possibility to control these DoFs independently according to their functionality. We have shown that the index can be valid for both prosthetic and robotic hands, dominant and nondominant hands. To define the index, the functionality of the different groups of DoFs of the hand (F/E, AB/AD, P.ARC, and T.OPP) was analyzed according to a human grasp experiment on twenty subjects with the four main GTs for personal autonomy in ADL. It was concluded that the relevance of the different groups of DoFs (*w*_*i*_) was 55% for F/E, 16% for AB/AD, 5% for P.ARC, and 24% for T.OPP. Thirteen artificial hands, including affordable 3D-printed prosthetic hands, advanced commercial prosthetic hands, and robotic hands, were evaluated and compared with the AIM, and the reason for their differences was discussed. The results obtained in this study should be taken into account in the concept design stage of new prototypes in order to obtain new designs that maximize their functionality. Further research will focus on new metrics for later design stages considering other design aspects (range of motion of the joints, relative length of the phalanges, orientation of the joints axes, etc.) and on experimental benchmarks to measure the grasping capability of artificial hands.

## Figures and Tables

**Table 1:** Joints and degrees of freedom (DoFs) of the human hand corresponding to the four different groups of DoFs defined.

Groups of DoFs	Joints and DoFs of the human hand
Finger flexion-extension (F/E)	MCP2_Flexion^∗^
	PIP2_Flexion^∗^
	DIP2_Flexion
	MCP3_Flexion^∗^
	PIP3_Flexion^∗^
	DIP3_Flexion
	MCP4_Flexion^∗^
	PIP4_Flexion^∗^
	DIP4_Flexion
	MCP5_Flexion^∗^
	PIP5_Flexion^∗^
	DIP5_Flexion

Finger abduction-adduction (AB/AD)	MCP2_Abduction^∗^
	MCP3_Abduction
	MCP4_Abduction^∗^
	MCP5_Abduction^∗^

Palmar arching (P.ARC)	CMC5_Flexion^∗^
	CMC4_Flexion

Thumb opposition (T.OPP)	CMC1_Flexion^∗^
	CMC1_Abduction^∗^
	MCP1_Flexion^∗^
	MCP1_Abduction
	IP1_Flexion^∗^

1: thumb, 2: index finger, 3: middle finger, 4: ring finger, 5: little finger; CMC: carpometacarpal joint, MCP: metacarpophalangeal joint, PIP: proximal interphalangeal joint, DIP: distal interphalangeal joint, IP: interphalangeal joint; ^∗^16 joint angles measured during the experiment with the CyberGlove.

**Table 2:** Classification of the DoF depending on the type of actuation and numeric coefficient associated.

Class	Type of actuation of the DoF	*c*
A	DoF actuated by one independent motor or actuator	1
B	DoF underactuated with other DoFs without a rigid coupling, allowing adaptive grasps (tendons, elastic elements)	0.75
C	DoF underactuated with other DoFs with a rigid coupling, not allowing adaptive grasp (linkages)	0.5
D	No actuation on the DoF, but passive motion allowed	0.25
E	DoF absent in the artificial hand	0

**Table 3:** Mean (SD) of the relative contribution *n*_*g*_ of the five PCs in each grasping task *T*_*g*_ (*g*: indicates the order of the tasks in Figure 2) and final value of the parameter *s*_*k*_ for each PC.

	PC1 (%)	PC2 (%)	PC3 (%)	PC4 (%)	PC5 (%)
*T*_01_	19.2 (6.4)	24.4 (7.6)	18.4 (7.7)	13.0 (7.7)	24.9 (9.7)
*T*_02_	22.6 (6.9)	14.2 (6.3)	25.1 (6.1)	22.4 (8.1)	15.7 (8.4)
*T*_03_	25.1 (4.7)	23.1 (4.7)	15.6 (4.3)	24.9 (8.9)	11.3 (5.5)
*T*_04_	33.5 (6.7)	19.1 (3.8)	12.6 (3.5)	12.1 (4.2)	22.7 (10.6)
*T*_05_	22.4 (8.3)	21.4 (8.9)	18.6 (8.5)	17.4 (10.6)	20.1 (13.9)
*T*_06_	28.2 (6.0)	21.0 (5.0)	17.9 (5.4)	17.0 (9.5)	16.0 (10.2)
*T*_07_	28.3 (5.0)	21.1 (3.8)	12.9 (4.3)	27.0 (9.2)	10.8 (5.8)
*T*_08_	39.1 (6.2)	23.6 (4.0)	12.9 (4.6)	11.8 (7.7)	12.5 (7.3)
*T*_09_	19.5 (8.2)	24.5 (7.1)	14.3 (7.9)	20.3 (10.1)	21.3 (9.0)
*T*_10_	30.9 (6.2)	18.6 (5.2)	20.1 (6.2)	9.6 (5.4)	20.7 (11.7)
*T*_11_	19.9 (3.4)	19.8 (7.7)	19.2 (7.9)	18.3 (10.8)	22.8 (13.3)
*T*_12_	34.0 (7.4)	20.4 (5.1)	12.8 (4.3)	12.1 (6.5)	20.8 (9.6)

*s*_*k*_	29.0	20.5	16.5	15.6	18.4

**Table 4:** Matrix *r*_*ik*_ and resulting *w*_*i*_ (equation (3)).

Groups of DoFs	Functional synergies					*w*_*i*_ (%)
	PC1 (%)	PC2 (%)	PC3 (%)	PC4 (%)	PC5 (%)	
Finger flexion-extension	79.6	50.7	42.5	51.8	34.9	55
Finger abduction-adduction	8.8	37.8	6.9	20.7	5.4	16
Palmar arching	4.4	3.4	15.3	0.2	1.9	5
Thumb opposition	7.2	8.1	35.3	27.3	57.9	24

**Table 5:** Parameter *k*_*i*_ (equation (2)) for each group of DoFs for the different artificial hands.

Artificial hand	F/E	AB/AD	P.ARC	T.OPP
IMMA	0.48	0.25	0	0.50
Cyborg Beast	0.29	0	0	0.18
Flexy-Hand	0.39	0.25	0	0.23
KIT	0.47	0	0	0.35
ADA	0.58	0.25	0	0.40
i-Limb	0.58	0	0	0.55
Bebionic	0.50	0	0	0.50
SensorHand	0.13	0	0	0.10
Michelangelo	0.13	0	0	0.30
FRH-4	0.46	0	0.50	0.40
Barrett	0.25	0.38	0	0.30
DLR/HIT II	0.83	1	0	0.70
Shadow	0.83	1	0.50	1

F/E: finger flexion-extension, AB/AD: finger abduction-adduction, P.ARC: palmar arching, T.OPP: thumb opposition.

**Table 6:** Results of the Anthropomorphism Index of Mobility (AIM) for different artificial hands and comparison with other indexes of the literature.

Artificial hand	AIM (%)	AI (%) [3]	A_R_ (%) [4]
IMMA	42		
Cyborg Beast	20		
Flexy-Hand	31		
KIT	34		
ADA	46		
i-Limb	45		
Bebionic	40		
SensorHand	10	0.25	
Michelangelo	14	2.80	
FRH-4	37	5.20	
Barret	27		10.38
DLR/HIT II	78		26.61
Shadow	88		39.93

**Table 7:** Range of motion of the hand joints (in degrees) obtained in the human grasp experiment.

	Thumb (°)				Index (°)			Middle (°)		Ring (°)			Little (°)		
	F	A	F	F	F	A	F	F	F	F	A	F	F	A	F
	CMC	CMC	MCP	IP	MCP	MCP	PIP	MCP	PIP	MCP	MCP	PIP	MCP	MCP	PIP
Min	-27	0	-24	-32	-22	-9	0	-16	0	-13	-7	-1	-13	-7	-2
Max	32	28	13	42	51	24	62	65	66	68	16	76	69	12	68
P5	-6	0	-10	-5	-4	-3	1	-1	1	-1	-1	1	-3	-1	0
P95	15	19	2	17	30	9	36	40	42	30	8	49	26	7	40

CMC: carpometacarpal joint, MCP: metacarpophalangeal joint, PIP: proximal interphalangeal joint, DIP: distal interphalangeal joint, IP: interphalangeal joint, F: flexion (+)/extension (-), A: abduction (+)/adduction (-), P: percentile.

## Data Availability

The human hand kinematics expressed as joint angles and scores referred to the five PCs, the loading matrix corresponding to these PCs, and the *c*_*ij*_ values for the joints of the artificial hands analyzed; the data used to support the findings of this study are included within the supplementary information files.

## Supplementary Materials

Supplementary Materials The excel file “JointAngles” contains the joint angles for each instant for the twelve tasks repeated three times by twenty subjects during the human grasp experiment. The excel file “Scores” contains the human hand kinematics in the human grasp experiment transformed to scores *f*_*tk*_ referred to five functional synergies for the twelve tasks repeated three times by twenty subjects. The excel file “LoadingsMatrix” contains the loadings *l*_*ijk*_ of the five functional synergies during ADL. The excel file “ArtificialHands” contains the value *c*_*ij*_ according to the method of actuation for each DoF in the artificial hands analyzed and the corresponding *k*_*i*_ of each hand.
